# The Digestive Diverticula in the Carnivorous Nudibranch, *Melibe leonina*, Do Not Contain Photosynthetic Symbionts

**DOI:** 10.1093/iob/obab015

**Published:** 2021-05-21

**Authors:** W H Watson, K M F Bourque, J R Sullivan, M Miller, A Buell, M G Kallins, N E Curtis, S K Pierce, E Blackman, S Urato, J M Newcomb

**Affiliations:** 1 Department of Biological Sciences, University of New Hampshire, Durham, NH 03824, USA; 2 Department of Pediatrics, Johns Hopkins Hospital, Baltimore, MD 21287, USA; 3 Department of Human Development and Family Studies, University of New Hampshire, Durham, NH 03824, USA; 4 Department of Psychiatry, Dartmouth College Geisel School of Medicine, Hanover, NH 03755, USA; 5 Department of Biology, Rollins College, Winter Park, FL 32789, USA; 6 Department of Biology, Ave Maria University, Ave Maria, FL 34142, USA; 7 Department of Integrative Biology, University of South Florida, Tampa, FL 33620, USA; 8 Department of Biology, University of Maryland, College Park, MD 20742, USA; 9 Department of Biology and Health Science, New England College, Henniker, NH 03242, USA; 10 College of Osteopathic Medicine, Lake Erie College of Osteopathic Medicine, Bradenton, FL 34211, USA

## Abstract

A number of nudibranchs, including *Melibe engeli* and *Melibe pilosa*, harbor symbiotic photosynthetic zooxanthellae. *Melibe leonina* spends most of its adult life on seagrass or kelp, capturing planktonic organisms in the water column with a large, tentacle-lined oral hood that brings food to its mouth. *M. leonina* also has an extensive network of digestive diverticula, located just beneath its translucent integument, that are typically filled with pigmented material likely derived from ingested food. Therefore, the focus of this project was to test the hypothesis that *M. leonina* accumulates symbiotic photosynthetic dinoflagellates in these diverticula. First, we conducted experiments to determine if *M. leonina* exhibits a preference for light, which would allow chloroplasts that it might be harboring to carry out photosynthesis. We found that most *M. leonina* preferred shaded areas and spent less time in direct sunlight. Second, we examined the small green circular structures in cells lining the digestive diverticula. Like chlorophyll, they exhibited autofluorescence when illuminated at 480 nm, and they were also about the same size as chloroplasts and symbiotic zooxanthellae. However, subsequent electron microscopy found no evidence of chloroplasts in the digestive diverticula of *M. leonina*; the structures exhibiting autofluorescence at 480 nm were most likely heterolysosomes, consistent with normal molluscan digestion. Third, we did not find evidence of altered oxygen consumption or production in *M. leonina* housed in different light conditions, suggesting the lack of any significant photosynthetic activity in sunlight. Fourth, we examined the contents of the diverticula, using HPLC, thin layer chromatography, and spectroscopy. The results of these studies indicate that the diverticula did not contain any chlorophyll, but rather harbored other pigments, such as astaxanthin, which likely came from crustaceans in their diet. Together, all of these data suggest that *M. leonina* does sequester pigments from its diet, but not for the purpose of symbiosis with photosynthetic zooxanthellae. Considering the translucent skin of *M. leonina*, the pigmented diverticula may instead provide camouflage.

## Spanish

Varios nudibranquios, incluidos *Melibe engeli* y *Melibe pilosa*, albergan zooxantelas fotosintéticas simbióticas. *Melibe leonina* pasa la mayoría de su vida adulta en pastos marinos o quelpo, donde captura organismos planctónicos en la columna de agua con una gran capucha oral forrada por tentáculos que llevan comida a su boca. *Melibe leonina* también tiene una extensa red de divertículos digestivos, ubicados justo debajo de su tegumento translúcido, que generalmente están llenos de material pigmentado probablemente derivado de alimentos ingeridos. Por lo tanto, el objetivo de este proyecto fue evaluar la hipótesis de que *M. leonina* acumula dinoflagelados fotosintéticos simbióticos en estos divertículos. Primero, realizamos experimentos para determinar si *M. leonina* se orienta hacia la luz, lo cual permitiría a los cloroplastos que podría albergar el realizar la fotosíntesis. Descubrimos que la mayoría de *M. leonina* prefería las áreas sombreadas y pasaba menos tiempo bajo la luz solar directa. En segundo lugar, examinamos las pequeñas estructuras circulares verdes en las células que recubren los divertículos digestivos. Al igual que la clorofila, exhibieron autofluorescencia cuando se iluminaban a 480 nm, y también tenían aproximadamente el mismo tamaño que los cloroplastos y las zooxantelas simbióticas. No obstante, la microscopía electrónica no produjo evidencia de cloroplastos en los divertículos digestivos de *M. leonina*. Es probable que las estructuras que exhibieron autofluorescencia en 480 nm fuesen heterolisosomas, lo cual es consistente con la digestión normal de moluscos. En tercer lugar, no encontramos evidencia de un consumo o producción de oxígeno alterado en *M. leonina* alojadas varias condiciones lumínicas, lo cual sugiere la ausencia de actividad fotosintética significativa en la presencia de luz solar. En cuarto lugar, examinamos el contenido de los divertículos mediante HPLC, cromatografía en capa fina, y espectroscopia. Los resultados de estos estudios indican que los divertículos no contenían clorofila, pero si otros pigmentos como la astaxantina que probablemente provenía de crustáceos en su dieta. Nuestros datos sugieren que *M. leonina* secuestra pigmentos de su dieta, pero no con el propósito de la simbiosis con zooxantelas fotosintéticas. Teniendo en cuenta la piel translúcida de *M. leonina*, los divertículos pigmentados podrían quizás proporcionar camuflaje.

## Introduction

Numerous animals have symbiotic relationships with photosynthetic organisms. One of the most well-known examples are the anthozoan corals that harbor dinoflagellates from the family Symbiodiniaceae, with the cnidarian providing a suitable environment and some metabolic waste product resources, while the dinoflagellates provide nutrients via photosynthesis (Baker 2003. A number of nudibranchs exhibit a similar symbiotic relationship with Symbiodiniaceae, including *Aeolidia papillosa* (McFarland and Muller-Parker 1993, *Aeolidiella alderi* (Marín and Ros 1991, *Baeolidia moebii* (formerly *Berghia major*; Kempf 1984, *Catriona maua* (Marín and Ros 1991, *Pteraeolidia ianthina* (Kempf 1984; Hoegh-Guldberg and Hinde 1986; Hoegh-Guldberg et al. 1986; Sutton and Hoegh-Guldberg 1990; Loh et al. 2006; Burghardt et al. 2008, *Spurilla neapolitana* (Marín and Ros 1991, and multiple members of the genera *Berghia* (Kempf 1991; Marín and Ros 1991; although see Monteiro et al. 2019, *Cuthona* (Marín and Ros 1991, and *Phyllodesmium* (Burghardt et al. 2008. All of these nudibranchs are members of the superfamily Aeolidoidea. In addition to the dinoflagellates, aeolids are also known for sequestering nematocysts from their cnidarian prey in cells lining their digestive diverticula, and these may provide some protection from predators (reviewed in Greenwood 1988, 2009; Putz et al. 2010; Goodheart and Bely 2017). Therefore, the anatomy of aeolids may foster symbiotic relationships with photosynthetic dinoflagellates.

That said, some non-aeolid nudibranchs also form symbioses with photosynthetic zooxanthellae, such as *Doto* (Marín and Ros 1991 and members of the genus *Melibe*. While it is thought that many species of *Melibe* may harbor Symbiodiniaceae (Gosliner and Smith 2003, only two species have been rigorously documented to form a symbiotic relationship with these zooxanthellae, *Melibeengeli* (Burghardt et al. 2008; Burghardt and Wägele 2014 and *Melibepilosa* (Kempf 1984. Unlike the aeolids noted above, *Melibe* do not obtain Symbiodiniaceae via their prey, but as bycatch from their unusual feeding method, which entails the opening and closing of a tentacle-lined oral hood that brings food to the radula-less mouth (Hurst 1968; Schuhmacher 1973; Ajeska and Nybakken 1976; Watson and Trimarchi 1992. In both *M. engeli* and *M. pilosa*, the Symbiodiniaceae are primarily housed in carrier cells that emanate from the digestive system, but also can be found in the digestive lumen itself and in the extracellular matrix below the epithelia of the cerata (Kempf 1984; Burghardt and Wägele 2014. Evidence suggests that the Symbiodiniaceae exhibit functional photosynthesis in some *Melibe*, based on increased growth and prolonged survival in light versus dark conditions (Kempf 1984; Burghardt and Wägele 2014.

*Melibe leonina* is another member of this genus and previous research has already demonstrated that light can have an influence on locomotion in this species (Newcomb et al. 2004; Newcomb et al. 2014. Specifically, *M. leonina* tend to both crawl and swim more at night. Morphological examination of a preserved specimen of *M. leonina* (Gosliner and Smith 2003 and a histological investigation of another specimen (Wägele and Johnsen 2001 suggest that *M. leonina* lacks photosynthetic symbionts. However, these studies only included data from a single specimen. Considering that other members of this genus do have a symbiotic relationship with photosynthetic zooxanthellae, a more thorough investigation of this possibility in *M. leonina* is warranted.

**Fig. 1:**
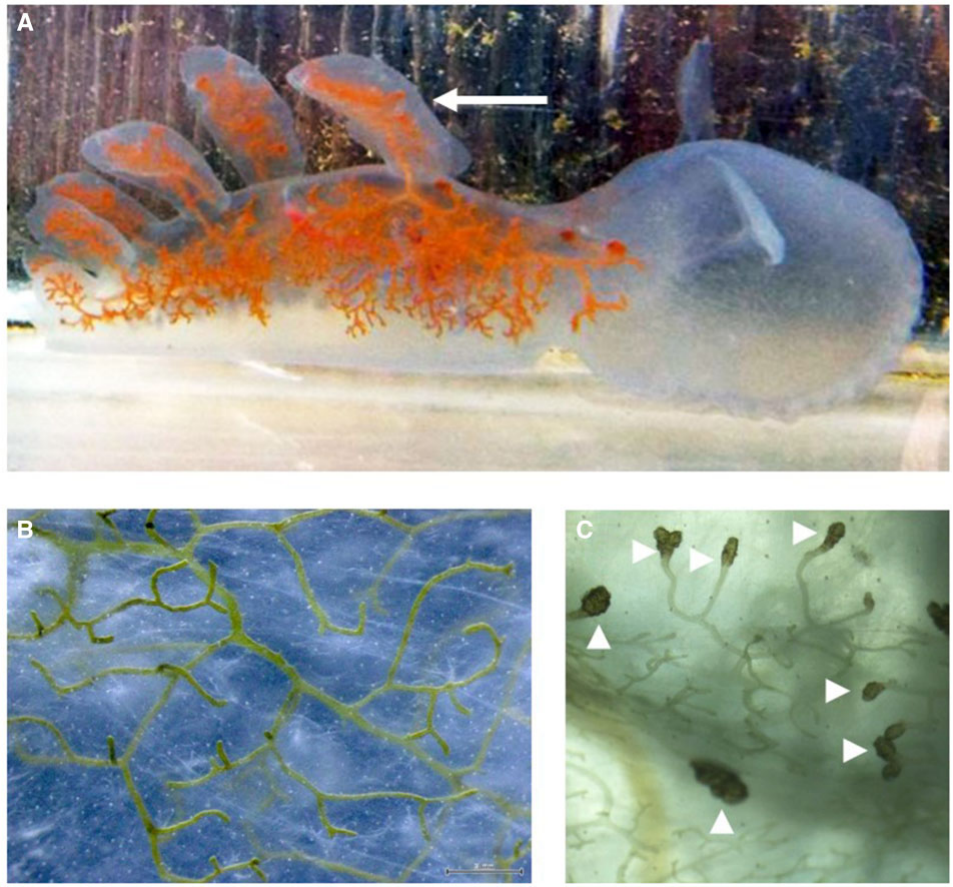
*Melibe leonina* digestive diverticula. (**A**) Image of a *M. leonina*, with anterior and the oral hood to the right. Diverticula (orange) emanate from the digestive system and propagate through a large portion of the animal, including the dorsal cerata (anterior most cerata indicated by white arrow). The diverticula vary in color between animals, but are typically orange, green, or brown, depending on their diet. This image is of an animal that had been in a flow-through seawater tank at Friday Harbor Laboratories, where it likely consumed dinoflagellates. (**B**) Close-up of the skin of a different animal, that had been captured from an eelgrass bed and held in a tank with eelgrass, showing extensive branching of green diverticula, right under the transparent epithelium. (**C**) Many of these diverticula end in tufts, just underneath the skin (white arrowheads).

*M.* *leonina* has translucent skin that covers a vast network of colored vessels, located just beneath the skin, that are branches of the digestive track (intestinal diverticula, Fig. 1). These vessels are sometimes orange or brown (Fig. 1A), but they are also often green (Fig. 1B and C) (Agersborg 1923. We hypothesized that, like some other *Melibe*, *M. leonina* may sequester zooxanthellae in its digestive diverticula to provide it with additional nutrients. In order to test this hypothesis we: (1) determined if, given a choice, *M. leonina* preferred to be in the light, which might aid in photosynthesis, or the dark; (2) used light and electron microscopy to investigate the presence of zooxanthellae or chloroplasts in cells associated with digestive diverticula; (3) measured oxygen consumption in the light versus the dark; and (4) examined the contents of the diverticula to determine if chlorophyll or other pigments were present. Data from all four sets of experiments suggest that, while the digestive diverticula do contain pigments from the diet, there is no evidence of functioning chloroplasts or a symbiotic relationship with photosynthetic dinoflagellates.

## Materials and methods

### Light–dark preference experiments

**Fig. 2:**
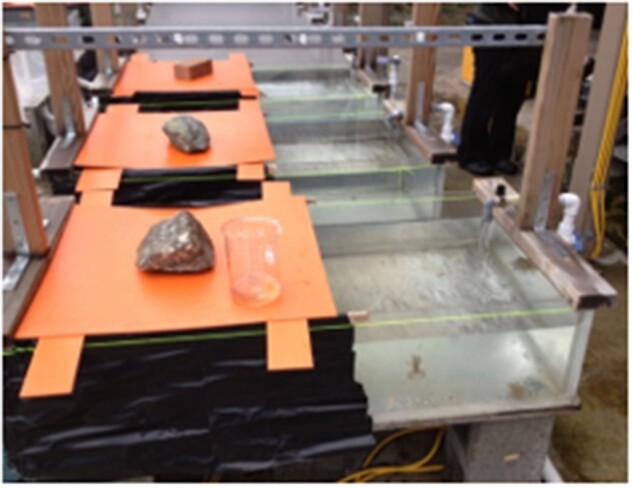
Experimental setups for behavioral experiments at Friday Harbor Laboratories. One half of the tank was covered with black plastic (left side in photo above), while the other side was exposed to sunlight during the day. Note that there were three replicate tanks.

The first of two light–dark preference experiments was carried out at the University of Washington’s Friday Harbor Laboratories (FHL), in the Puget Sound, WA. Specimens of *M. leonina* were collected from an eelgrass bed located in Parks Bay, Shaw Island. Three rectangular tanks, approximately 1.0 × 0.5 m, with a continuous flow of ambient seawater, were partially covered with black plastic and a wooden covering so that one side was shaded and the other side was exposed to natural sunlight (Fig. 2). For each of three trials, five *M. leonina* (3 tanks × 5 animal × 3 trials = a total of 45 animals) were placed in each of the tanks, where they remained for 6 days. The location of each animal (shaded or lit region) was recorded at four time points: 8 am, 12 pm, 4 pm, and 8 pm. From these data, we calculated the percent of animals on the side of the tank that was not shaded (i.e., was exposed to daylight) at each time point. Averages between the four time points were statistically compared with an ANOVA and a *posthoc* Tukey’s test. In select experimental tanks, a HOBO data logger was used to record temperature and light levels on the uncovered side of the tank during the course of the trial. A control trial was also done once, with five animals in each of the three tanks (a total of 15 animals). The control trial followed the same procedure, but the tanks were completely covered with black plastic. In this case, we briefly opened the black plastic at each time point to determine the location of animals.

The second experiment was conducted at the University of New Hampshire (UNH; Durham, NH), using *M. leonina* that were collected from Puget Sound and shipped to UNH. Animals were housed in an aquarium containing natural seawater, that was located inside a coldroom held at 15°C, with a 12 h:12 h light:dark cycle. A 40-L aquarium filled with chilled seawater was placed on a windowsill, so that it would receive natural illumination in the daytime. Black paper covered half of all four sides, and the top, yielding a choice tank similar, but smaller, to the one used at FHL. The temperature of the water in the aquarium was held at 15°C with a cooling coil connected to a recirculating chiller.

In these UNH experiments, only a single *M. leonina* was placed in the aquarium at a time and its behavior was continuously recorded for 3–5 days (mean of 67 h of data was obtained per animal, *n* = 11) using a camera connected to an ADC digitizer, that transmitted images to a Macintosh iBook. Gawker software was used to create a time-lapse digital video, with frames obtained once per second. During analysis, time-lapse videos were played back at 15 frames/s. In the daytime, animals were scored as being on the dark (left) side, light (right) side, or in the transition area between these light conditions. In the night, they were only scored as being on the left side or right side.

### Light, fluorescence, and electron microscopy

Small pieces of the diverticula of four *M. leonina* that had recently been shipped from the west coast were removed and placed on a slide with filtered seawater and covered with a coverslip. They were then viewed and photographed both with a compound light microscope (Nikon Axioplan), and a Zeiss LSM 880 confocal microscope using an excitation wavelength of 480 nm, which typically causes chlorophyll to emit red light.

For electron microscopy, specimens (*n* = 4) were fixed, within 2 h of collection, in 2% (v/v) glutaraldehyde in cacodylate buffer (0.15 M cacodylate containing 0.58 M sucrose, pH 7.5) followed by three 10 min washes in cacodylate buffer. To target digestive diverticula cells, cerata were removed from the animals and post-fixed with 2% (w/v) osmium tetroxide in the same buffer for 1 h, and then stained in 2% (w/v) uranyl acetate for 1 h. Dehydration consisted of 10 min washes in an ethanol series (35–100%), followed by three 10 min washes in propylene oxide. Next, small pieces (3 × 4 mm) of cerata were dissected and infiltrated with three 60 min washes in propylene oxide: Araldite–Epon resin (1:1, 1:2, and 1:3 v/v, in sequence) (Araldite 502/Embed 812, Electron Microscopy Sciences). Samples were then submerged in 100% Araldite–Epon resin for 60 min, embedded in fresh Araldite–Epon, and incubated for 12 h at 60°C until cured.

Thin sections (60–90 nm) were cut with an ultra-microtome (M2T-B, Sorvall Porter-Blum) and placed on 200 µm mesh, uncoated copper grids. Sections were stained for 1 min with 0.01 M lead citrate, followed by 1 min with 2% uranyl acetate, and then again with lead citrate for 1 min; they were then rinsed a final time with double-distilled water. Sections were viewed and photographed at the Electron Microscope Core Facility of the Department of Integrative Biology at the University of South Florida (Tampa, FL) using a transmission electron microscope (TEM) (Morgagni 268D, FEI).

### Oxygen consumption

The working hypothesis we were testing with these experiments was that if *M. leonina* diverticula contained functional chloroplasts, then oxygen consumption/production should be altered in light versus dark conditions. This experiment was carried out both with intact animals, and with sections of the diverticula that were removed and placed in a small amount of seawater. In the intact animal experiments, either individual *M. leonina*, or groups of six animals (a total of 38 animals), were placed in 500 mL containers and allowed 30 min to settle down. The containers were kept at 15°C by partially submerging them in 15°C seawater. The experiment commenced when the rubber stopper used for the lid was closed. A Vernier oxygen electrode (Beaverton, OR), that was connected to a Vernier Labquest, was inserted through a hole in the rubber lid to monitor changes in the oxygen content over time. Changes in oxygen levels (mg O_2_/L) were collected for 1 h in the light (transparent container) and 1 h in the dark (container painted black), and the order was randomized. The experiments with the diverticula were conducted in the same manner, except the container only had a volume of 20 mL. Mass-specific metabolic rate (MR) was calculated as follows: MR = oxygen consumption (mg/L/h) × volume (L) × 1 mL/1 mg × 1/mass (g).

### Identification of pigments

In order to determine the types of pigments that were present in the *M. leonina* diverticula, pigments were extracted and then separated with high-performance liquid chromatography (HPLC), as well as thin layer chromatography (TLC). For HPLC, pigments from one individual that had been held in an aquarium with access to kelp were extracted in 100% acetone overnight (1:1000 concentration of tissue to acetone) and separated using a HPLC Agilent1100 B.103 ChemStation. For comparison, pigments were also extracted from kelp, using the same methods.

For TLC, diverticula (∼1 mL) were dissected out of the cerata of *M. leonina* that had either been housed with brine shrimp nauplii (*Artemia parthenogenetica*) or kelp (*Macrocystis* from FHL or *Alaria* from the Gulf of Maine), and diluted 1:25 in 100% acetone. Similar quantities of kelp, brine shrimp, and astaxanthin (Nature Made) were also used for comparison samples. In each case, the tissue was mechanically homogenized with a small plastic pestle and incubated for 24 h at 4°C. One milliliter of petroleum ether was added and mixed by inversion, followed by 4 mL of 5% sodium chloride. The pigment layer formed at the top of the solution, which was removed and placed into a centrifuge tube. Sodium sulfate was slowly added, to remove excess water. The final pigment sample was then stored at −20°C, until needed for TLC. For these experiments, pigment samples were applied near the bottom of a silica TLC plate and placed in a beaker with a small volume of mobile phase, consisting of 60% hexane, 30% ethyl acetate, and 10% triethylamine. Pigments were separated as the mobile phase wicked up the plates.

For spectroscopy, separated pigments from animals that had been housed with either kelp or *Artemia* were dissolved back into acetone, and the absorbance spectrum, from 350 to 750 nm (25-nm increments), was measured using a Cary 5E UV–Vis–NIR spectrophotometer.

## Results

### Light–dark preference experiments

**Fig. 3:**
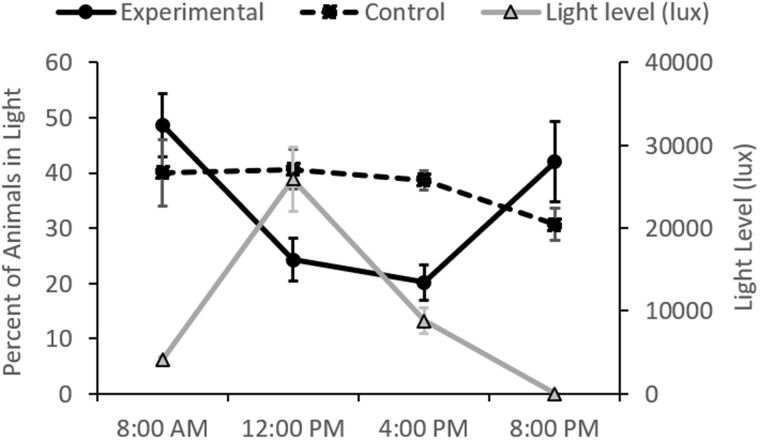
In experimental trials (solid line, left axis), *M. leonina* preferred shaded areas of the tank when light levels (gray line and right axis) were highest, at midday and late afternoon. This preference was significantly different from the 8 am time point (*P* = 0.0003), when animals were almost equally divided between dark and light areas. In control experiments (dotted line, left axis), the location of animals did not significantly differ between time points. Data points are averages ± standard error.

In the experiments conducted outside at FHL, more than 75% of the experimental animals (*n* = 45) preferred to be in the shaded portion of the tank during midday and afternoon, when the sunlight was the brightest (Fig. 3). This was significantly different from the early morning and early evening (*P *=* *0.0003, df = 3, *F* = 9.0), when they were distributed fairly evenly between the dark and light portions of the tanks. In contrast, control animals (*n* = 15) that were in tanks that were covered so there was no light gradient, did not change their behavior much throughout the day, spending about 40% of their time in the half of the tank that was lit in the experimental trials and about 60% of their time on the other side, regardless of the time of day (*P *=* *0.3, df = 3, *F* = 1.4). The control animals may not have spent half of their time on each side of the tank, because the flow-through seawater traveled from the “lit” side to the “shaded” side. Light levels on the exposed side of the tank ranged between an average of 8780–25,888 lux during periods when animals preferred the shaded region (4 pm and noon, respectively). In contrast, light levels averaged 4090 lux or less in the early morning and evening, when animals did not exhibit a preference for a tank region.

**Fig. 4:**
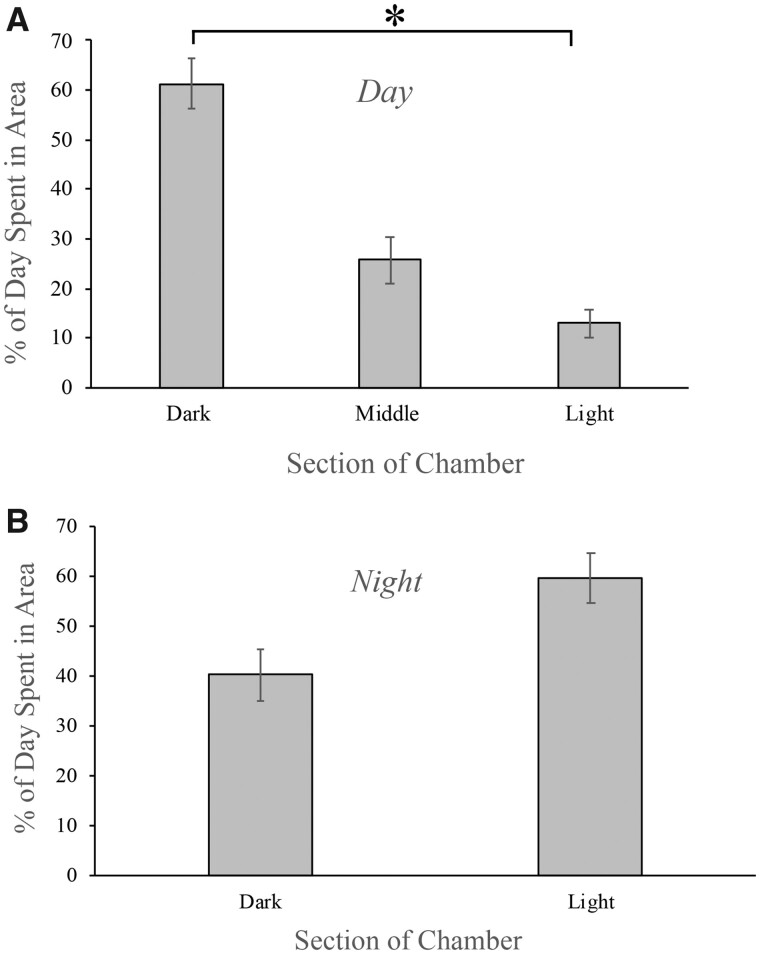
Avoidance of bright light by individual *M. leonina* in an aquarium illuminated by sunlight. (**A**) In the daytime, *M. leonina* (*n* = 11) spent significantly more time in the shaded (dark) region of the aquarium, than the illuminated side (light; *P* = 0.009 [indicated by asterisk]). The percent of time spent in the shaded region was not quite significantly different than the percent of time spent at the border between the shaded and lit regions (middle; *P* = 0.06). (**B**) However, at night, there was no significant difference in regard to the amount of time animals spent on each side of the aquarium (*P* = 0.059). Error bars indicate standard error.

In the choice experiments at UNH, the animals also preferred to be on the shaded side of the tank for significantly more time than the illuminated side during the daytime (Fig. 4A;*P *=* *0.009, ANOVA with Tukey’s post-test, *n* = 11). On a sunny day, the light levels on the illuminated side of the tank would reach 30,000–45,000 lux. The avoidance of bright light became quite evident when reviewing the videos because, as the sun changed position in the sky and thus illuminated different areas of the clear side of the aquarium, the animals would change their position in an apparent effort to avoid the brighter light (Supplementary Video S1). There was also a preference for the shade versus the middle area, but this difference was not quite significant (*P *=* *0.059). In comparison, there was no significant difference in where animals were found in the tank during the night (Fig. 4B;*t*-test, *P *=* *0.90).

### Light, fluorescence, and electron microscopy

**Fig. 5:**
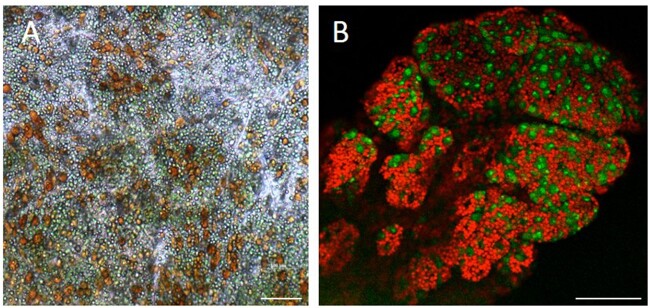
Micrographs of cells in diverticula. (**A**) Light micrograph of cells lining the distal tubule of a digestive diverticulum. Some cells are faint green, suggestive of chlorophyll, while others are dark orange or brown. (**B**) Confocal microscopy image of cells in a tuft at the end of the digestive diverticulum in a ceras. The red and green colors represent two different wavelengths of autofluorescence emitted in response to 480 nm excitation. The red structures are consistent in size (2–7 μm across) and autofluorescence response with chloroplasts or zooxanthellae. Scale bars = 100 μm.

Light microscopy (*n* = 4) revealed small circular vesicles in cells lining digestive diverticula (Fig. 5A). Many of these vesicles were ∼10 µm in diameter and were dark orange in color. Others were green and slightly smaller (2–7 µm in diameter), consistent in size with chloroplasts or zooxanthellae (Kempf 1984; Curtis et al. 2010. When illuminated with 480 nm-wavelength light, these smaller green vesicles emitted red light (Fig. 5B), similar to chlorophyll (Takahashi 2019. The larger orange vesicles emitted a green wavelength when excited at 480 nm.

**Fig. 6:**
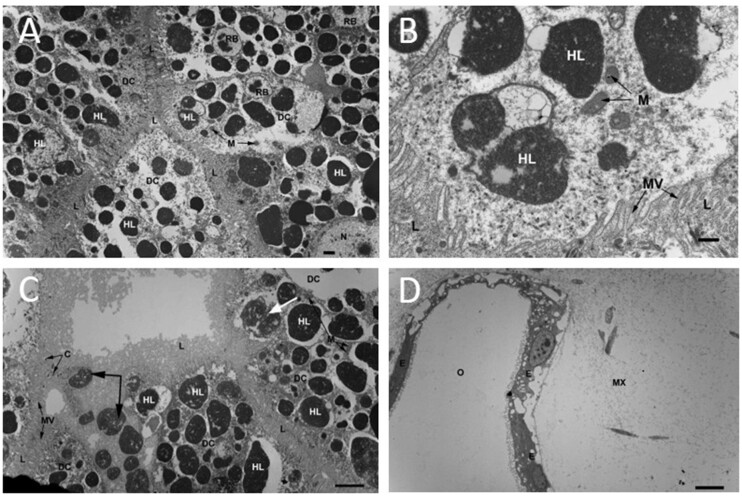
Transmission electron microscopy (TEM) did not reveal the presence of chloroplasts or zooxanthellae. (**A**) TEM of cells lining the digestive diverticulum in a ceras. The cells contained numerous heterolysosomes (HL) and other phagosome-like structures typical of molluscan digestion. No photosynthetic structures were seen. HL, heterolysosome; L, digestive tubule lumen; DC, digestive cell; M, mitochondria; N, nucleus; RB, residual body. Scale bar = 5 μm. (**B**) TEM of heterolysosomes contained in cells lining the digestive diverticulum. These structures were likely food particles, which had been recently phagocytized and fused with lysosomes and/or peroxisomes. They were roughly the same size as the structures seen in Fig. 5, and just as numerous. It is therefore likely that these heterolysosomes were the structures emitting autofluorescence (Fig. 5), as there was no evidence of any photosynthetic structures, such as thylakoids. HL, heterolysosome; L, digestive tubule lumen; MV, microvilli; M, mitochondria. Scale bar = 1 μm. (**C**) Another TEM of cells lining the digestive diverticulum in a ceras. This image shows putative foodstuffs (large unlabeled arrow) lying inside the digestive tubule lumen (L), most likely about to be phagocytized. L, digestive tubule lumen; HL, heterolysosome; MV, microvilli; DC, digestive cell; C, cilia; M, mitochondria. Scale bar = 5 μm. (**D**) Free edge of a ceras lacking zooxanthellae. In species containing zooxanthellae, the dinoflagellates are often present just below the epithelium. O, free space outside of slug tissue; MX, sparsely cellulated matrix which comprises the portion of the ceras between the epithelium and the digestive diverticulum. Scale bar = 5 μm.

TEM of cells lining the digestive diverticula in cerata (*n* = 4) indicated that these cells contained many vesicles similar in size and number to the green vesicles in the light micrographs of whole diverticula (Fig. 6A–C). However, these appeared to be heterolysosomes and other phagosome-like structures typical of molluscan digestion. There was no evidence of any photosynthetic structures, such as thylakoids, in any of the TEM sections. In gastropods containing zooxanthellae, the dinoflagellates are often present just below the epithelium, in the extracellular matrix between the outer tissue layer and the digestive diverticula. In *M. leonina*, there was no evidence of any zooxanthellae in these regions (Fig. 6D).

### Oxygen consumption

To determine if potential zooxanthellae or harbored chloroplasts could produce oxygen via photosynthesis in the light, oxygen consumption was compared for animals in both the light and dark. Our working hypothesis was that as the *M. leonina* consumed the oxygen in the closed container, oxygen levels would decrease, but the rate of decrease might not be as large in the light, as in the dark, because the symbionts would be replacing some of the oxygen due to photosynthesis. However, there was no significant difference in the MR of animals in the dark, compared with those in the light (two-tailed unpaired *t*-test, *P *=* *0.78). The negative slope of oxygen consumption over time was also not significantly different (data not shown).

### Identification of pigments

**Fig. 7:**
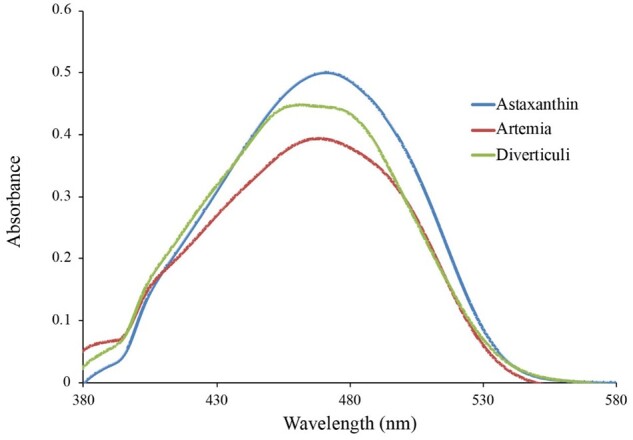
Absorbance spectra for the pigment astaxanthin and extracts from *Artemia* and *M. leonina* diverticula match well, with similar peaks around 470 nm. These data suggest that the pigment in the diverticula is likely due to the fact that the *M. leonina* were fed *Artemia* nauplii in the laboratory, and that these crustacean prey contain the pigment astaxanthin. In their native habitat, *M. leonina* are also likely to consume zooplankton that contain an astaxanthin-like pigment.

HPLC revealed a peak for chlorophyll *a* in the pigments extracted from kelp, whereas this peak was not present in samples extracted from *M. leonina* diverticula, even for animals located on kelp while feeding. In contrast, diverticula samples from *M. leonina* fed on *Artemia* had a small cluster of peaks located where astaxanthin should appear, whereas kelp samples did not contain this cluster. TLC also indicated the presence of chlorophyll *a* in kelp extract, both in samples from kelp collected in NH and in WA, near FHL. Similar to HPLC, TLC did not indicate the presence of chlorophyll *a* in samples extracted from *M. leonina* diverticula. Instead, diverticula from animals fed on *Artemia* contained an orange pigment with a retention factor (Rf) value of 0.7–0.8, similar to pigments extracted from *Artemia* nauplii. Spectroscopy indicated that the absorbance curves for pigment extracted from *Artemia* nauplii and *M. leonina* diverticula were similar to the curve for astaxanthin (Fig. 7).

## Discussion

Our working hypothesis, given its translucent skin, the presence of pigmented material in the digestive diverticula, and the presence of symbionts in other species in the same genera, was that *M. leonina* would harbor photosynthetic zooxanthellae. However, none of the experiments we conducted supported this hypothesis. First, *M. leonina* did not express positive phototaxis that has been observed in many invertebrates that have symbiotic relationships with photosynthetic zooxanthellae, including cnidarians (Pearse 1974; Yamashiro and Nishira 1995; Foo et al. 2020, flatworms (Serȏdio et al. 2011; Nissen et al. 2015, and sea slugs (Rahat and Monselise 1979; Gallop et al. 1980; Weaver and Clark 1981. In some animals, phototaxis is dependent upon the presence of symbiotic zooxanthellae (Pearse 1974; Foo et al. 2020. In others, positive phototaxis may occur, but only at lower intensities of light (Gallop et al. 1980; Weaver and Clark 1981; Serȏdio et al. 2011), possibly because in photosynthetic organisms, excess light can be damaging (Haeder et al. 1995; Cruz et al. 2013. In fact, the sacoglossan *Elysia timida* will shade its kleptoplasts (“stolen” chloroplasts) by closing its parapodia (Rahat and Monselise 1979; Jesus et al. 2010, and another sacoglossan, *Plakobranchus* cf. *ianthobaptus*, suffers greater weight loss and reduced kleptoplast photosynthetic activity when in high light treatments, when compared with low or moderate light conditions (Donohoo et al. 2020. Therefore, it is possible that *M. leonina* avoided bright light in our study either because animals did not harbor symbiotic dinoflagellates or because the intensity of light was too high.

Individuals exposed to light also did not exhibit a change in their rate of oxygen consumption, which we hypothesized would be the case if animals harbored algae that carried out photosynthesis in sunlight and produced oxygen as a byproduct. This held true for both intact animals and isolated tissue (cerata with digestive diverticula). Preliminary pulse amplitude fluorometry experiments (not reported here due to small sample sizes) also did not find any evidence of photosynthetic activity. In slugs that engage in kleptoplasty, such as *Elysia*, oxygen production is positively correlated with light irradiance (Rumpho et al. 2000; Giménez-Casalduero and Muniain 2006 and algal density (Hoegh-Guldberg and Hinde 1986. Therefore, the lack of such correlation in our study provides further evidence that these animals did not harbor photosynthetic zooxanthellae.

While initial results from light and fluorescence microscopy suggested the possible presence of chloroplasts or zooxanthellae in the digestive diverticula of *M. leonina*, electron microscopy did not provide any evidence of photosynthetic structures. Instead, the TEM data suggest that the structures of interest in light/fluorescence microscopy resembled heterolysosomes, residual bodies, and other features consistent with normal molluscan intracellular digestion (Graves et al. 1979; Steneck and Watling 1982; Kress et al. 1994. These results are also consistent with RNA sequencing data for *M. leonina*, which have yet to find evidence of chloroplasts or zooxanthellae (Goodheart et al. 2015; Cook et al. 2018.

We also failed to identify any chlorophyll in extracts of *M. leonina* diverticula, even though we did succeed in extracting other pigments, which matched those present in its food source. Animals fed *Artemia* harbored a pigment with a Rf value and absorbance spectrum similar to astaxanthin, the pigment in *Artemia* that gives it an orange color. It is possible that a higher concentration of Symbiodiniaceae in the water would result in the presence of chlorophyll in digestive diverticula, although this would not necessarily mean that *M. leonina* had a symbiotic relationship with it. Digestive diverticula in *M. leonina* can appear in different colors (Fig. 1; Agersborg 1923 and it is likely that this difference is the result of diet. The sacoglossan *Elysia crispata* (ecotype *clarki*) is known to vary in color due to variations in diet, and another sacoglossan, *Placida kingstoni*, is known to turn green after feeding on the alga *Bryopsis plumosa* (Curtis et al. 2010. However, *P. kingstoni* immediately digests the chloroplasts and does not retain them, and therefore a green coloration does not always imply a symbiotic relationship.

The symbiotic relationship with zooxanthellae that is exhibited by many nudibranchs, including *M. engeli* and *M. pilosa*, is different than the kleptoplasty (i.e., stolen chloroplasts) exhibited by a number of sacoglossans (Christa et al. 2015, and even protists (Cevasco et al. 2015, dinoflagellates (Gast et al. 2007), and plants (Krause 2015. The former often results, though not always, in a mutually beneficial relationship. In contrast, kleptoplasty involves the digestion of the algae and incorporation of chloroplasts into host cells (Pierce and Curtis 2012; Pierce et al. 2015. To date, there is no evidence that nudibranchs have evolved kleptoplasty.

Based on the data from this study, we conclude that *M. leonina* does not require a symbiotic relationship with photosynthetic zooxanthellae. This is in contrast to previous studies involving two other members of this genus, *M. engeli* (Burghardt et al. 2008; Burghardt and Wägele 2014 and *M. pilosa* (Kempf 1984. In lieu of a required symbiotic relationship with zooxanthellae, there may be other reasons that a transparent integument and pigmented diverticula may be advantageous. For example, it may help animals blend into their surroundings (Marín and Ros 1991; Di Marzo et al. 1993; Wägele 2004, or help in protecting underlying tissues from damage due to ultraviolet radiation, by either blocking the damaging wavelengths of light or aiding recovery due to the high antioxidant properties of some pigments, such as astaxanthin (Davinelli et al. 2018). Pigmented diverticula invading the cerata may simply be a means of increasing surface area for metabolically active tissue to have easy access to the epithelial surface for efficient gas exchange. Of course, none of these explanations are mutually exclusive and further studies are recommended in order to determine all of the advantages of having an extensive network of pigmented digestive diverticula located immediately below translucent skin.

## Supplementary Material

obab015_Supplementary_Data
